# Corrigendum

**DOI:** 10.1002/prp2.889

**Published:** 2021-10-23

The authors of Young et al. (2020)^1^ have supplied the following correction to their article.

An error in the SAS code used has led to calculating incorrect marginal odds ratios in the article.

On page 7 of the Supporting Information (Appendix A: SAS code, The MCMC procedure), the correct SAS code used to calculate a marginal odds ratio is:

$$\text{MargOR}=\left(\text{ProbTreat}/\left(1-\text{ProbTreat}\right)\right)/\left(\text{ProbCntrl}/\left(1-\text{ProbCntrl}\right)\right);$$

This resulted in errors in the calculation of the marginal odds ratios in Table 2 and in the Supporting Information. The correct odds ratios are provided below:

**Table:** 

Treatment assignment	Main model	Linear spline for HCV viral load	Any substance use rather than recent	Main model	Main model
Treatment success	Main definition	Main definition	Main definition	Restricted	Expanded
Odds ratio	0.67 (0.39–1.1)	0.70 (0.39–1.2)	0.66 (0.37–1.1)	0.66 (0.30–1.4)	0.95 (0.59–1.6)

On page 3 of the Supporting Information, the correct odds ratio is 0.67 (95% credible interval 0.39–1.14).

The authors apologize for this error and state that the conclusions of their study are not affected.
